# Terrestrial molluscs of Pemba Island, Zanzibar, Tanzania, and its status as an “oceanic” island

**DOI:** 10.3897/zookeys.70.762

**Published:** 2010-11-29

**Authors:** B Rowson, B. H. Warren, C. F. Ngereza

**Affiliations:** Biodiversity & Systematic Biology, National Museum of Wales, Cathays Park, Cardiff, CF10 3NP, UK; UMR C53 PVBMT, Université de La Réunion - CIRAD, 7 chemin de l’IRAT, Ligne Paradis, 97410 St. Pierre, Réunion, France; National Museums of Tanzania, Shabaan Robert Street, PO Box. 511, Dar es Salaam, Tanzania

**Keywords:** Land-snails, Stylommatophora, Pulmonata, Caenogastropoda

## Abstract

Pemba is thought to have had a longer and/or stronger history of isolation than its better-known counterpart, Unguja. The extent to which the biota support this hypothesis of greater oceanicity have been debated. Here, Pemba’s terrestrial mollusc (“land-snail”) fauna is surveyed and reviewed for the first time. We find at best equivocal evidence for the following hallmarks of greater oceanicity: impoverishment, imbalance, and a high rate of endemism. At least 49 species are present, families are represented in typical proportions, and there are only between two and four island-endemic species - i.e. a 4% to 8% rate of endemism. For land-snails, isolation thus seems to have been short (Pleistocene) or, if longer, weak. Nevertheless, Pemba does host endemic and globally rare species. Forty-five percent of the species found, including most of these, is restricted to forest reserves, with Ngezi Forest Reserve particularly rich. A further 45% are able to tolerate the island’s woody cultivated habitats. One new snail species (Cyclophoridae: Cyathopoma) and one new slug species (Urocyclidae: Dendrolimax pro tem.) are described. New data and illustrations are provided for other taxa.

## Introduction

**Figure 1.**
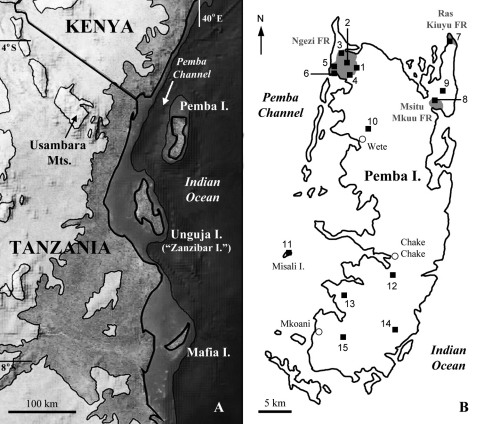
**A** Pemba and the surrounding area. Contours: 200m and 1000m (above sea level); 200m (below sea level). The land below 400m, including the islands, roughly corresponds to the Zanzibar-Inhambane vegetation mosaic of White (1983) in which coastal forest fragments are scattered. **B** sites surveyed on Pemba in 2009, numbered as in Table 1, with forest reserves (shaded areas) and large towns marked.

Pemba is one of the two main Indian Ocean islands of Zanzibar, Tanzania, the other being Unguja (itself commonly referred to as “Zanzibar”). It has long been recognised that although the two are of comparable size, topography, distance from the mainland, as well as climate and climatic history (e.g. Clarke 2000), they differ in their geological and biotic history. Geologically, both islands consist of Miocene rocks of continental origin fringed by uplifted Pleistocene coral rag limestone platforms (Kent et al. 1971, Schlüter 1997, Mukuza et al. 2002). However, the channel separating Pemba from the mainland and from Unguja reaches approximately 800m depth while that between Unguja and the mainland is less than 200m; Pemba effectively lies off the continental shelf (Fig. 1A). While the presence of a terrestrial fauna in Pemban rocks supports the presence of land since at least the middle Miocene (Pickford 2008), this land is believed to have been part of the mainland until the formation of the Pemba Channel by a graben fault approximately 6 Ma (early Pliocene; Kent et al. 1971, Clarke and Burgess 2000). Other geological evidence has suggested an even earlier separation in the late Miocene (see Moreau and Pakenham 1940, Stockley 1942, Pickford 2008). Sea level lowstands of up to 145m below present since Pemba’s separation, while critical for the evolution of other western Indian Ocean island faunas (e.g. Peake 1972, Warren et al. 2010), would therefore not have sufficed to reconnect Pemba to the mainland or Unguja. Unguja, in contrast, was most recently isolated from the mainland in the Pleistocene (Stockley 1942, Clarke and Burgess 2000) probably as little as 10–18 thousand years ago. Thus even if Pemba’s isolation was as recent as the latest Pliocene (1 Ma), it would have remained an island for up to 100 times longer than Unguja.

A corollary of the theory of island biogeography is that the hallmarks of greater “oceanicity” (= isolation, sensu Peake [1972]) would then be expected in Pemba’s biota relative to that of Unguja: a depauperate fauna skewed towards vagile species and with a greater degree of endemism. Which of these features the fauna (which is imperfectly known and certainly modified by man) shows has been the subject of debate. The fauna of both islands, plus that of the Tanzanian island of Mafia, is on the whole derived from the adjacent mainland (e.g. Voeltzkow 1923, Moreau and Pakenham 1940, Burgess et al. 1998). Pemba was found to be an important sub-centre of endemism for the region by Burgess et al. (1998), the only one of the three islands to be so, largely as a result of high faunal endemism. As Beentje (1990) and Baker and Baker (2002) suspected, further endemics have been discovered in other groups, sometimes reported to have a Mascarene affinity (e.g. Nahonyo et al. 2005, Dijkstra et al. 2007). Addressing the vertebrate fauna of the islands, Moreau and Pakenham (1940) noted that Pemba’s fauna was particularly impoverished and unbalanced, and showed Mascarene affinities not present in Unguja. However, they described endemism as low and noted that these “peculiarities” of Pemba were shown mainly by volant species. They probably had in mind the endemic Pemba Flying Fox Pteropus voeltzkowi, Pemba Scops-owl Otus pembaensis, Pemba White-eye Zosterops vaughani, Pemba Sunbird Nectarinia pembae and perhaps the Pemba Green Pigeon Treron pembaensis. Peake (1971) gave both Pteropus and Otus as examples of western Indian Ocean island lineages found only upon islands even where they were close to continents, whose presence would impart an “oceanic facies”, i.e. appearance to faunas. Both Zosterops and Nectarinia, and pigeons generally, show Indian Ocean radiations that could also be included in this category (Warren et al. 2003, 2006). Moreau and Pakenham (1940) considered the high vagility of these taxa to reduce their importance, and concluded from the vertebrate fauna that Pemba could not have been isolated much longer than Unguja or Mafia, implying a Pleistocene separation. The geological evidence for a Pliocene separation was however, restated (Stockley 1942, Kent et al. 1971) and the debate remains unresolved (Pickford 2008). Of course, endemism among such vagile taxa could equally be interpreted as indicating stronger, not weaker, isolation, especially from the mainland. Indeed, Moreau and Pakenham (1940) considered dispersal between the mainland and Pemba particularly difficult because of currents passing through the Pemba Channel. However, currents would be predicted to have the same effect when considering dispersal between the mainland and Unguja. The prevailing wind seasonally alternates in direction (Baker and Baker 2002, Pickford 2008). It seems that Moreau and Pakenham (1940) were arguing for a short but strong period of isolation for Pemba. If correct, Pemba’s fauna should be a close relative of the mainland’s, in which any impoverishment and imbalance results mainly from extinction. A long period of strong isolation would instead result in an unequivocally oceanic fauna in which impoverishment and imbalance results both from extinction and a low rate of immigration due to dispersal limitation. Endemism in either case should be high relative to that of Unguja, which is thought to have experienced a much shorter period of isolation (that may also have been weaker).

Here we discuss results of the first survey of Pemba’s terrestrial mollusc fauna (“land-snails”, including slugs) in this context. In principle, land-snails are much less vagile than volant animals, yet there is abundant evidence for long-distance dispersal to islands (e.g. Gittenberger 2007). In East Africa, the lowland land-snail fauna is poorly-known but now documented in a few coastal forest fragments in which endemism to fragments is high (Tattersfield 1998, Lange and Mwinzi 2003, Rowson 2007). These coastal forests form a region of endemism scattered through the Zanzibar-Inhambane vegetation mosaic of White (1983) and support the majority of the region’s narrow-range endemics (Burgess et al. 1998, Clarke and Burgess 2000) which include land-snails (Verdcourt 2000, Rowson 2007). Emberton et al. (1997) found that within Tanzania, both diversity and endemism peaked in the northern coastal forests, those nearest Pemba. The forest on Pemba is greatly depleted outside three small government forest reserves (FRs) (Beentje 1990, Burgess et al. 1998, Baker and Baker 2002). Ngezi FR is the best-known of these. Beentje (1990) drew attention to its mixture of plant species with coastal, montane, Asian and Madagascan affinities. Secondary woodland or thicket (especially clove plantations, often abandoned) covers much of the rest of the island and constitutes intervening or ecotonal habitat between FRs. The land-snail fauna of Pemba has scarcely been published upon prior to this study. Voeltzkow (1923: pp.172, 179, 185) recorded 10 species in a general account of Pemba’s natural history; Haas (1929) examined Voeltzkow’s material, localising some records and adding an 11th species. (Verdcourt (1983, 2000, 2006) did not repeat all these records in his abbreviated checklists for East Africa but did add two other species, making 13 in total. This contrasts with at least 58 species now known from Unguja (Rowson 2007). Our aim was to obtain a more accurate land-snail inventory for Pemba and to clarify its affinities to the mainland and other islands.

## Materials and methods

To examine how the land-snail fauna reflects the strength and duration of Pemba’s isolation we sought evidence of a) impoverishment; b) imbalance in composition; and c) increased endemism, each relative to Unguja and to mainland coastal forests. This required that as many species as possible were documented. We also aimed to clarify patterns of species presence or absence between habitat types and between FRs, data on which are currently absent for most Pemban taxa and limited for other Tanzanian coastal taxa.

Survey work was carried out in February 2009. Survey sites were selected in each FR and in additional sites covering most of the island (plus Misali I., a small island nature reserve to the west; Fig. 1B, Table 1). The highest point on Pemba is 95m above sea level so all were at roughly equivalent altitude, but were in different vegetation types and on different underlying soil or bedrock (especially at Ngezi FR, which encloses a complex of different forest types [Beentje 1990]). Survey methodology was a combination of direct search and litter sieving adapted from Tattersfield (1996); collecting effort was quantified although it varied across sites. While no survey can guarantee to find all species, these are two of the most important considerations in surveys of this type (Cameron and Pokryszko 2005). Land-snails were identified with reference to collections and the literature and are deposited at the National Museum of Wales, UK (NMW) and National Museums of Tanzania (NMT). As in Rowson (2007), informal morphospecies names (“sp. A” etc.) are avoided, one advantage being more accurate comparison with other studies.

**Table 1.** Survey sites (numbered as in Fig. 1B) and grouped into habitat types. Ngezi FR sites are grouped into two habitat types according to underlying geology. “Person-hours” is the total time spent on direct search and “litter” is the approximate volume of litter sieved (litres). Codes in square brackets are original site names and dates of collection.

Details				Search effort	
No.	Habitat type	Latitude / longitude	Description	Person-hours / litter	Habitat type total
1	Ngezi FR (sand/soil)	-4.939691 / 39.708538	High moist forest on sandy alluvial soil within reserve less than 1km from entrance [N1,7.2.09]	10 / 8	28 / 104
2	-4.935586 / 39.699225	High moist forest on sandy alluvial soil in centre of reserve [N3,8.2.09]	12 / 64
3	-4.919125 / 39.695277	High forest and swamp forest on dark alluvial soil in north of reserve [N6,11.2.09]	3 / 8
4	-4.962009 / 39.706607	High moist forest on muddy alluvial soil in stream valley in south of reserve [N7,11.2.09]	3 / 24
5	Ngezi FR (coral rag)	-4.946789 / 39.678755	Dry forest on dark, sandy soil on coral rag on Tondooni peninsula within reserve [N2,7.2.09]	12 / 24	22 / 64
6	-4.959658 / 39.685578	Dry forest and thicket on dark, sandy soil on coral rag on coast of Tondooni peninsula within reserve [N4,8.2.09]	10 / 40
7	Ras Kiuyu FR	-4.907837 / 39.86269	Dry forest on light, not sandy soil on coral rag in degraded high forest in south of reserve [R1,10.2.09]	10 / 32	10 /32
8	Msitu Mkuu FR	-5.000081 / 39.832091	Moist forest on dark, not sandy soil on coral rag in high forest in north of reserve [M1,10.2.09]	12.5 / 32	12.5 / 32
9	Non-FR sites	-4.967589 / 39.855051	Mango trees on reddish, sandy soil near Kiuyu School [K1,10.2.09]	1.5 / 0	26.5 / 48
10	-5.042864 / 39.73521	Clove and fruit tree woodland on reddish, not sandy soil near Wete [117,11/13.2.09]	6 / 16
11	-5.235746 / 39.606019	Mature bushland on coral rag in interior of Misali Island [MI,14.2.09]	3 / 0
12	-5.276008 / 39.769607	Clove and fruit tree woodland on dark, not sandy soil near Matuleni [MT,15.2.09]	3 / 8
13	-5.313227 / 39.689677	Clove and fruit tree woodland on reddish, not sandy soil near Wambaa [119,13.2.09]	6 / 0
14	-5.387244 / 39.765766	Clove and fruit tree woodland on dark, sandy soil near Chwaka [CH,15.2.09]	3 / 16
15	-5.379286 / 39.691308	Clove and fruit tree woodland on reddish, not sandy soil near Mtondoni [MO,15.2.09]	4 / 8

## Results

### a) Impoverishment and community diversity

4261 mollusc individuals representing forty-seven species were found in total, with only two previously recorded slug species not refound (Table 2). For each habitat type and FR we recorded approximately 16–68 times as many individuals as species, exceeding Cameron and Pokryszko’s (2005) suggested sampling minimum of ten times, so we should have a good first estimate of total richness and its variation. Among habitat types, Ngezi FR (sand/soil) (27 species) and Ngezi FR (coral rag) (23 species) were richer than Msitu Mkuu FR (23 species) or Ras Kiuyu FR (14 species). When both habitat types at Ngezi FR were combined, 33 species in total were recorded; four were found only on sand/soil and six only on coral rag. Although our survey cannot accurately compare abundances (Cameron and Pokryszko 2005) the total number of individuals found at Ngezi was much lower on sand/soil (436) than on the coral rag (1138), despite greater collecting effort (Table 1). When aggregated, non-forest sites hosted the greatest number of species (30) but with a mean of only 11.7 species per site, indicating substantial between-site heterogeneity.

**Table 2.** Species recorded from Pemba. An entry in the “Unguja” column indicates the same species occurs on Unguja (page numbers and figures in Rowson, 2007); “nf” in the final column indicates two previously recorded species, both slugs, not found during the present survey.

*Species*				*Recorded for sites*															
*Species*	*Note*	*Figs.*	**Unguja** (pp. / figs.)	Ngezi FR (sand/soil)				Ngezi FR (coral rag)		Ras Kiuyu FR	Msitu Mkuu FR	Non-FR sites							*No. sites*
				*1*	*2*	*3*	*4*	*5*	*6*	*7*	*8*	*9*	*10*	*11*	*12*	*13*	*14*	*15*	
Assimineidae																			
“Assiminea” aurifera Preston, 1912	3	2	429 / 18		+	+		+	+	+	+	+		+	+		+		10
Cyclophoridae																			
Cyathopoma azaniense Verdcourt, 1978	4	13–15							+										1
Cyathopoma pembense sp. n.	1	16–27									+		+		+	+			4
Pomatiidae																			
Tropidophora zanguebarica (Petit, 1850)	5	3	432 / 3		+			+	+	+	+		+	+	+	+	+	+	11
Veronicellidae																			
Laevicaulis alte (Férussac, 1821)	6		432				+												1
Cerastidae																			
Gittenedouardia conulina (von Martens, 1869)	7	29	433 / 27		+			+	+						+				4
Rachis punctata (Anton, 1839)			434 / 29									+							1
Rhachistia braunsi (von Martens, 1869)	8	28	434 / 31						+	+	+			+			+		5
Nesopupidae																			
Nesopupa minutalis (Morelet, 1881)			433 / 9								+								1
Chondrinidae																			
Gastrocopta klunzingeri (Jickeli, 1873)			433 / 12							+									1
Succineidae																			
Quickia concisa (Morelet, 1848)			447 / 20	+															1
Euconulidae																			
Afroguppya quadrisculpta (Connolly, 1939)		45–47							+						+				2
Afropunctum seminium (Morelet, 1873)		48–50	445						+	+									2
Microcystina minima (H. Adams, 1867)	9	51–53	445 / 14							+									1
Helicarionidae																			
Kaliella barrakporensis (L. Pfeiffer, 1854)		42–44	446	+			+	+	+		+		+		+				7
Ariophantidae																			
Sitala jenynsi (L. Pfeiffer, 1845)	10	36–38	446												+				1
Urocyclidae																			
Pembatoxon insulare van Goethem, 1975	11		447 / 40	+	+						+								3
Trichotoxon heynemanni Simroth, 1888	11																		nf
Elisolimax roebucki (Simroth, 1910)	11																		nf
“Dendrolimax” vangoethemi sp. n.	2	11–12, 64–75			+				+	+									3
Thapsia curvatula von Martens, 1897	12		446 / 4	+	+														2
Thapsia insulsa Preston, 1910	13	39–41												+			+		2
Trochonanina mozambicensis (L. Pfeiffer, 1855)	14	9	438 / 43	+	+		+	+	+		+	+		+	+	+	+	+	12
Ferussaciidae																			
Cecilioides callipeplum (Connolly, 1923)			435 / 21	+	+				+		+								4
Achatinidae																			
Achatina (Lissachatina) allisa Reeve, 1849	15		438 / 43	+	+		+	+	+	+			+		+			+	9
Achatina (Lissachatina) fulica hamillei Petit, 1859	16		438 / 45								+					+			2
Subulinidae																			
Allopeas gracile (Hutton, 1834)	17		436 / 26			+					+	+		+			+		5
Curvella subvirescens (E. A. Smith, 1890)	18	30		+	+														2
Opeas delicatum Taylor, 1877	19		436 / 23–24		+			+	+	+	+	+	+		+	+	+		10
Opeas lamoense Melvill & Ponsonby, 1892	20		436 / 22	+		+					+	+	+		+	+	+		8
Pseudoglessula (Kempioconcha) subolivacea agg. (E. A. Smith, 1890)			436 / 36	+	+	+	+	+	+	+	+		+	+	+	+		+	13
Pseudopeas igembiense Connolly, 1923		32	435–436 / 17					+	+										2
Striosubulina striatella (Rang, 1831)	21	31																+	1
Subulina intermedia Taylor, 1877			435 / 25	+	+	+	+	+	+		+		+		+	+			10
Subulina octona (Bruguière, 1789)	22		435 / 32			+					+		+		+	+	+	+	7
Subulona ordinaria Preston, 1910	23	4						+	+		+		+						4
Streptaxidae																			
Edentulina obesa (Taylor, 1877)	24	8	439 / 41	+	+		+	+	+				+			+			7
Gonaxis (Gonaxis) denticulatus (Dohrn, 1878)	25	5, 54–56	440 / 38	+	+	+	+	+	+		+		+		+	+	+	+	12
Tayloria shimbiensis Connolly, 1923	26	7, 33–35		+	+	+	+				+		+						6
Streptostele (Raffraya) acicula (Morelet, 1877)			444 / 47–48		+	+		+	+	+	+		+			+			8
“Gulella” (Aenigmigulella) aenigmatica (E. A. Smith, 1890)	27	57–59									+		+		+				3
“Gulella” peakei van Bruggen, 1975			441–442 / 54						+										1
“Gulella” radius (Preston, 1910)	28	60–61			+			+	+	+	+		+		+	+	+		9
Gulella baccata (Preston, 1913)			440 / 57						+										1
Gulella gwendolinae (Preston, 1910)	29	62	452	+						+				+	+				4
Gulella jod (Preston, 1910)			441 / 50	+	+		+		+	+									5
Gulella planidens (von Martens, 1892)	30	63			+	+					+				+		+		5
Gulella sexdentata (von Martens, 1869)			442 / 58													+			1
Gulella streptostelopsis van Bruggen, 2007			442 / 52						+										1
*Total species for site*				16	20	10	10	15	25	14	23	6	16	8	19	14	12	7	
*Total species for FR / habitat type*				27				25		14	23	30							
*Total species for Pemba*				47 (+ 2 nf ) = 49															

All FRs and habitat types contained at least one species not recorded elsewhere on Pemba. Importantly for conservation, 21 species (approximately 45% of the 47 species found) were found only in FRs, including the 10 rarest species (those represented by the fewest individuals) and all the slugs found. For example, Curvella disparilis and Thapsia curvatula were found only at Ngezi FR (sand/soil); Microcystina minima was found only at Ras Kiuyu FR; and Nesopupa minutalis was found only at Msitu Mkuu FR. Another 21 species (45%) were found in both FRs and non-forest habitat types, including the 10 most abundant species. These include several taxa treated by Verdcourt (2000) as forest specialists (Tayloria shimbiensis, Opeas delicatum, Subulona ordinaria) and at least one previously unrecorded from forest habitats (Assiminea aurifera). Both the Pemba-endemic Cyathopoma pembense and the Eastern Arc species “Gulella” aenigmatica occurred only in Msitu Mkuu FR and in non-forest sites. The remaining five species (approximately 10%) were found only in non-forest habitats. These include the only certainly introduced species (Striosubulina striatella) as well as two further taxa treated by Verdcourt (2000) as forest specialists (Thapsia insulsa and Gulella sexdentata).

### b) Imbalance

In overall species richness, Pemba’s fauna is dominated by the families Streptaxidae (13 species, 27%), Subulinidae (10, 20%), and Urocyclidae (7, 14%). The order remains the same if Achatinidae are included in Subulinidae. Individually, Ras Kiuyu FR has Euconulidae, and non-forest sites have Cerastidae in third place instead of Urocyclidae, but this may be due to the difficulties of sampling slugs. The Maizaniidae are the only conspicuous coastal forest family that appear to be reliably absent from Pemba.

### c) Endemism and affinities

The following three species (6% of the total of 49) are known only from Pemba and we consider them endemic: Cyathopoma pembense, Dendrolimax vangoethemi, and Elisolimax roebucki. There are no endemic genera or subgenera and all three endemics have close relatives both on the mainland and elsewhere in the western Indian Ocean. Dendrolimax vangoethemi probably occurs in the Usambaras (see below) while Elisolimax roebucki has had doubts raised over its species status (see Rowson 2007 p. 447). The populations of “Gulella” radius on Pemba may be considered a separate species (see Notes, 28). Thus the rate of species endemism could be as low as 2% (considering only Cyathopoma pembense endemic) or as high as 8% if (considering Cyathopoma pembense, both slugs, and the Pemba “Gulella” radius to be endemic). Accepting a 6% rate, a total of 36 (73%) of Pemba’s species are also found on Unguja. Of these, 33 also occur on the mainland, sometimes in small areas. The remaining two species (4% of Pemba’s total) are known only from Pemba and Unguja: Pembatoxon insulare and Gittenedouardia conulina. There are doubts about the species status of the latter (see Notes, 7).

### Descriptions of new taxa

Museum abbreviations are as follows: BMNH: Natural History Museum, London, UK; IRSNB, Royal Belgian Institute of Natural Science, Brussels, Belgium; MNHN, Muséum national d’Histoire naturelle, Paris, France; RMNH, Naturalis, Leiden, the Netherlands; NMT, National Museums of Tanzania, Dar-es-Salaam, Tanzania; NMW, National Museum of Wales, Cardiff, UK; NMSA, Natal Museum, Pietermaritzburg, South Africa; ZMB, Museum für Naturkunde, Berlin, Germany.

#### 1. Cyathopoma pembense

Rowson sp. n.

urn:lsid:zoobank.org:act:9D732594-3393-417A-9F66-F1459AC232BB

Figs 16 27 

##### Type material:

(all from TANZANIA: Zanzibar: Pemba Island). **Holotype** (NMW.Z.2009.013.00001): adult shell stored dry; in leaf litter, near Wete (Locality 10 in Fig. 1 and Table 1), 13 February 2009, leg. B. Rowson, B. H. Warren & C. F. Ngereza. Paratypes (NMW.Z.2009.013.00002-00032): 31 adults and juveniles in 80% ethanol; other data as holotype. Paratypes (NMW.Z.2009.013.00033-00077): 45 adults and juveniles stored dry; other data as holotype. Paratypes (NMW.Z.2009.013.00078-00079), 2 adults gold-coated for SEM; other data as holotype. Paratypes (NMT): 2 adults stored dry; collection data as holotype. Paratypes (BMNH.20100582): 1adult & 1 juvenile stored dry; collection data as holotype. Paratypes (MNHN): 1 adult & 1 juvenile stored dry; collection data as holotype. Paratypes (NMSA.L8207/T2591): 2 adults stored dry; collection data as holotype. Paratypes (RMNH): 2 adults stored dry; collection data as holotype. Paratypes (NMW.Z.2009.013.00080-00096): 17 adults and juveniles in 80% ethanol; in leaf litter, Msitu Mkuu FR (Locality 8 in Fig. 1 and Table 1), 10 February 2009, leg. B. Rowson, B. H. Warren, C. F. Ngereza & paid local collectors. Paratypes (NMW.Z.2009.013.00097-00174): 78 adults and juveniles stored dry; other data as previous. Paratype (NMW.Z.2009.013.00175): 1 adult gold-coated for SEM; other data as previous. Paratypes (NMW.Z.2009.013.00175-00176): 2 adults in 80% ethanol; in leaf litter, near Matuleni (Locality 12 in Fig. 1 and Table 1), 15 February 2009, leg. B. Rowson & C. F. Ngereza. Paratypes (NMW.Z.2009.013.00177-00210): 33 adults and juveniles stored dry; other data as previous. Paratype (NMW.Z.2009.013.00211): 1 adult stored dry; in leaf litter, near Wambaa (Locality 13 in Fig. 1 and Table 1), 13 February 2009, leg. B. Rowson, B. H. Warren, C. F. Ngereza & paid local collectors.

##### Diagnosis:

Shell relatively large (to 4.20mm wide) and strongly depressed. When fresh, with spirally-ridged operculum and characteristic periostracum of radial lamellae peripherally extended into long hairs gathered into points, or much shorter hairs gathered into fringes. When denuded, with relatively few spiral keels.

**Description of holotype:** Adult shell (Figs 16–18) relatively large for the genus in Africa, 2.25mm x 3.95mm including periostracum, strongly depressed, of approximately 4.5 regularly expanding whorls, with wide, perspective umbilicus. Peristome effectively complete, slightly thickened and flaring, especially basally. Aperture and operculum effectively circular. Operculum calcareous, outer surface with multispiral, blade-like raised lamella of approximately 9 revolutions, weakly convex as a result; inner surface smooth. Protoconch smooth, with irregular malleation discernible only at extreme magnification (Fig. 25). Teleoconch periostracum of fine, extremely close (<0.025 mm apart) radial lamellae, running from suture to suture. Lamellae each prolonged into long, flat periostracal extensions (“hairs”) extending well beyond the whorl periphery, forming spiral keels (up to four on the body whorl), with less-pronounced periostracal keels continuing into umbilicus. Periostracal hairs (in life and in fresh shells, whether wet or dried) regularly gathered at their tips to form bunches of six or more hairs (Fig. 25).

##### Further description from paratypes:

The periostracum of C. *pembense* forms a continuum of variation. At one extreme are individuals with hairs gathered together at their tips (as in the holotype). At the other are those in which the periostracal lamellae form instead a rough, raised periostracal fringe where the lamellae appear cemented together (Figs 19–21; 27). These extremes are more frequent than intermediate forms, but such intermediates do occur, in which the bunches of hairs are irregularly missing, probably worn away (see below). The two extreme forms are sympatric at three of the species’ four localities – i.e., at the type locality, at Msitu Mkuu FR, and near Matuleni (Localities 10, 8 & 12 respectively in Fig. 1 and Table 1). At the fourth locality near Wambaa (Locality 13 in Fig. 1 and Table 1), only one individual was found, and was of the fringed form. Both forms include both live- and dead-collected individuals, and both adult and juvenile shells. The size ranges overlap, although the fringed form seems to reach a slightly larger maximum (4.00–4.20mm wide with 4.25–4.5 whorls). Other features of the shell (shape, operculum, and protoconch; Fig. 26) are consistent across all individuals. Shells from which the periostracum has been lost were common but always empty, and cannot be assigned to either form. Such denuded shells (Figs 22–24) have relatively few (up to 8) weak spiral keels on the body whorl (including umbilical part), with fine, extremely close incised radial lines between the keels. All nine live-collected individuals of the fringed form were dissected and a penis was detected in six of them. A penis was not detected in any of nine individuals of the hairy form.

##### Remarks:

This species is attributed to Cyclophoridae: Cyathopoma *sensu lato* following Emberton (2003). All forms of Cyathopoma pembense differ from the few other East African Cyclophoridae in being larger and more depressed than Cyathopoma azaniense Verdcourt, 1978 (Figs 13–15), an undescribed *azaniense*-like species from the East Usambaras (Verdcourt 2006; NMW material examined), and the Malawian Cyathopoma tres van Bruggen, 2008 (van Bruggen 2008). They are also larger than the Central African Cyathopoma papillaris (von Martens, 1892) and have fewer spiral keels (see van Bruggen 1986). The elaborate periostracum appears to be unique among East African taxa but similar features occur in some southeast African and western Indian Ocean island taxa. Cyathopoma pembense differs from species of the southeast African Chondrocyclus Ancey, 1898 either in the operculum or in periostracal features; Cyathopoma putealis Connolly, 1939 and Cyathopoma trifimbriatus Connolly, 1939 have fringes like Cyathopoma pembense but very different opercula (see van Bruggen 1986; Herbert and Kilburn 2004). Cyathopoma pembense is more depressed and differs in periostracum from the Seychelles Cyathopoma blanfordi H. Adams, 1868 (see Gerlach 2006a, b). Photographs of Cyathopoma pembense were compared with the BMNH types of several Comoros species attributed to “Cyclotopsis” (Cyathopoma nevilli Morelet, 1877, Cyathopoma ilicum Morelet, 1877, and Cyathopoma horrida Morelet, 1887). Although worn, none of these were an exact match for Cyathopoma pembense. Nor does it agree with the descriptions or figures of any other cyclophorid of the Comoros (see Fischer-Piette and Vukadinovic 1974), Madagascar (Emberton 2003, 2004), the Mascarenes (Griffiths and Florens 2006), the Seychelles (Gerlach 2006a, b), nor any Asian species known to us.

The variation shown by this species is striking. One might consider the extreme periostracal forms separate species, albeit indistinguishable when the shells are denuded. However the presence of intermediates suggests that this is not the case. The variation could result from sexual dimorphism (hairy forms female, fringed forms male) which would explain their occurrence in sympatry. However, sexual dimorphism would not explain the existence of intermediate forms. It would also demand that the three fringed individuals without a penis were immature males rather than females, when equally possible is that fringed forms consist of three females and six males while and all nine hairy individuals were immature. Natural wear and corrosion on the periostracum, presumably from hairy to fringed forms, would explain the latter possibility and account for the continuum of variation. It would not, however, easily explain the existence of live animals of both types (in each case both adults and juveniles in sympatry, where presumably the whole population is exposed to similar factors causing wear and corrosion. Possibly both sexual dimorphism and wear on the shells play a part in this unusual pattern. More speculatively, other alternatives include incomplete speciation or hybridisation between two closely related species.

##### Distribution:

Apparently endemic to Pemba island.

##### Etymology:

*pembense*, from Pemba island, a noun in the generative case.

#### 2. "Dendrolimax" vangoethemi

Rowson sp. n.

urn:lsid:zoobank.org:act:40EB98A1-E93A-47FA-B81C-C4F545FFD298

Figs 11–12 64–75 

##### Type material

(all from TANZANIA: Zanzibar: Pemba Island). **Holotype** (NMW.Z.2009.013.00211): slug 30.0 mm long in 80% ethanol, on understorey foliage during day, Ngezi FR (Locality 2 in Fig. 1 and Table 1), 8 February 2009, leg. B. Rowson, B. H. Warren, C. F. Ngereza & local collectors.

Paratype 1 (NMW.Z.2009.013.00212): slug 37.5 mm long in 80% ethanol; other data as holotype. Paratype 2 (NMW.Z.2009.013.00213): slug 31.0 mm long in 80% ethanol; other data as holotype. Paratype 3 (NMW.Z.2009.013.00214): slug 25.0 mm long in 80% ethanol; other data as holotype. Paratype 4 (IRSNB.IG.31599/MT2317): slug 18.0 mm long in 80% ethanol; other data as holotype. Paratype 5 (NMT): slug 19.0 mm long in 80% ethanol, on understorey foliage during day, Ngezi FR (Locality 6 in Fig. 1 and Table 1), 8 February 2009, leg. B. Rowson, B. H. Warren, C. F. Ngereza & local collectors. Paratype 6 (NMW.Z.2009.013.00215): slug 18.5 mm long in 80% ethanol; leaf litter during day, Ras Kiuyu FR (Locality 7 in Fig. 1 and Table 1), leg. B. Rowson, B. H. Warren, C. F. Ngereza & local collectors.

##### Diagnosis:

Medium-sized slug (to at least 55mm in life) with strong keel prolonged into long caudal appendage, with mantle completely covering shell. Pale to colourless, with dorsum covered in pustules. Viscera not extending far into tail, shell mineralised, jaw with no or weak projection. Radula unique in having up to 280 tiny, tricuspid teeth per half-row. Genitalia broadly similar to other Dendrolimax.

##### Description:

Note: points of agreement with an unnamed East Usambara species as discussed by Verdcourt and Polhill (1961) (see below) are marked with “*”.

External features: Medium-sized slug (extended length to at least 55mm in life, or 37.5mm in 80% ethanol)*. Tail strongly keeled* and hollowed out behind mantle*; keel prolonged into long straight caudal appendage* above small caudal pore*. Viscera extending little more than half-way into tail*. Body bell-shaped in cross-section when extended, but able to flatten body considerably. Sole not narrow, evenly tripartite*. Peripodial grooves clear, from tail to genital orifice and head. Mantle fully attached posteriorly, free anteriorly, not grooved, subangulate rather than rounded behind, completely covering shell, lacking a dorsal pore or slit*. Pneumostome in posterior third of mantle*. Genital orifice far forward, near right lower tentacle. No head wart or similar structure detected. Integumental tubercles barely detectable on mantle, tail or cephalopodium; instead, whole dorsum rather densely and regularly covered in hemispherical (rather than prickly) pustules*. Dorsum largely colourless and translucent*, with green, grey or pink tinge*, acquiring a green cast when on foliage; keel white; sole colourless. Diffuse, slate-grey pigment on caudal appendage, bordering keel* and/or in obscure blotches or bands on mantle in some specimens*, absent in others; remains on preservation. Pustules conspicuously white*, remaining so on preservation. Ommatophore retractors grey-ochre on preservation.

Jaw and radula: Jaw solid, semi-lunate, lacking median projection (holotype), or projection very weak (paratype). Radula of holotype broad (3.65 mm wide × 2.20 mm long), of 155 rows (over 50 angular rows per mm length). Teeth extremely small* and extremely numerous*, to nearly 450 in each half-row*, with a central tooth. All teeth (including central tooth) tricuspid*, with very little change across the row, perhaps becoming more s-shaped laterally. Ectocones larger (or at least projecting further) than mesocones in all teeth except central tooth. This is unlike any radula figured in van Goethem (1977) where mesocones are always the largest cusps, and the maximum number of teeth per half-row is around 280, and only in radulae over 5.00 mm wide. The radula form may suggest a microphagous, rather than phytophagous diet.

Shell and pallial complex: Shell unguiform, bilaterally symmetrical, to at least 4.10 mm long, infilled, mineralised and white* (i.e. not fragile as in other Dendrolimax). Pallial area well vascularised.

Genitalia: Right ommatophore retractor passes between penis and vagina. No atrial diverticulum or stimulator*. Long flagellum present in place of calc sac*. Epiphallus long, stiff, not spiralling around penis*. Penial retractor short, attaching well below flagellum, perhaps obtaining from diaphragm. Penis with basal sheath, contiguous with penis wall apically, internally with longitudinal pilasters and a basal papilla; similar to that of several Dendrolimax (see van Goethem 1977). Bursa copulatrix duct arising low on vagina, bound to it by sheath-like circular muscle fibres. Bursa copulatrix long, weakly clavate, reaching upper part of spermoviduct*. Vagina and free oviduct with a clear, thick-walled sheath*. Hermaphroditic duct extremely short*, barely perceptible between spermoviduct and large yellow ovotestis, which lies near rear of mantle. Albumen gland small, hook-shaped*. No spermatophores were recovered from the Pemba material which may not be fully adult.

##### Remarks:

This distinctive species was found only in FRs. At Ngezi, the slugs were found on the underside of large understorey leaves, up to 2m above ground. The body was held flattened with one optic tentacle protruding (Fig. 12). At Ras Kiuyu FR the species was found in litter. It appears to be undescribed although it (or a similar species) may occur in the East Usambara Mts. Beyond that its affinities are less certain.

Van Goethem (1977) thoroughly revised the known urocyclid slugs of Africa and Madagascar and provided keys to internal characters. The Pemba species keys readily out to Dendrolimacini (sole genus Dendrolimax Heynemann, 1868). The only East African record of the mainly Central-West African Dendrolimax is an unnamed and incompletely described species collected by Verdcourt (1960) from Thika Gorge, Kenya, who indicated a swollen lower oviduct not present in the Pemba species. Moreover the Pemba species differs from all other Dendrolimax in the radula, body form, and shell, although the genitalia are similar. It shows much clearer similarities to a taxon referred to as “*Genus et species nov*.” by Verdcourt and Polhill (1961, p. 32–33, fig. 42) from Sigi in the East Usambara Mts. (1961). They said, “This mollusc does not belong to the family Urocyclidae judging by [the] radula but to an isolated subfamily of the Helicarionidae near to the Durgellinae”. Van Goethem (1977) could not obtain material of this taxon for his revision but considered it a urocyclid. He treated it as “Species E”, *incertae sedis* after Dendrolimacini and Upembellini along with a “Species D” from Grand Comore to which he noted a similarity in the genitalia, but not the radula. Neither Verdcourt and Polhill (1961) nor van Goethem (1977) were certain whether the specimens of “Species E” or “Species D” were adult. This is an important consideration since slugs may change in appearance as they grow. However, neither Verdcourt and Polhill, van Goethem with his experience of growth series of many taxa, nor other slug workers (e.g. Forcart 1967) could attribute these forms to any known species or genus. Verdcourt and Polhill (1961) noted that there was no absolute criterion, e.g. concerning the size of the albumen gland, for recognising adulthood in urocyclid slugs. Since 1977 there has been little further work on the group in East Africa. The Pemba species, which will probably prove to include also the East Usambara species, is here described provisionally in Dendrolimax. Consideration was given to erecting a new genus but owing to a lack of unique features in the genitalia, and the large number of available genus-group names, is avoided until more data are available.

Although this species is fully limacised, there are also similarities in the body form and genitalia to numerous Afrotropical semi-slug genera, among them Verrucarion van Mol, 1970 of West and central Africa (van Mol 1970) and Malagarion Tillier, 1979 of Madagascar. The resemblance to the latter extends to the radula and white pustules (see Tillier 1979, Emberton 1994). Van Mol (1970) treated all African genera in Urocyclidae: Urocyclinae or Gymnarioninae. Tillier (1979) considered Malagarion to belong, with the Mascarene Colparion Laidlaw, 1938 in Helicarionidae: Ariophantinae and not Urocyclinae(/idae). He noted that the radula, but not genitalia, of Malagarion was similar to the Asian Durgellini (founded on the Burmese semi-slug Durgella Blanford, 1863). Verdcourt and Polhill (1961) had also noted a radula similarity between their East Usambara species and the Durgellinae, although this was not discussed by van Goethem (1977). Certainly the radula would be unique in Urocyclinae sensu van Goethem (1977), and resembles Malagarion in the size and number of teeth, and the large ectocones (at least on the more marginal teeth of Malagarion). The monophyly of these major groups is questionable while the systematics of tropical Limacoidea is still far from resolved (e.g. Tillier 1979, Hausdorf 1998, Schileyko 2002), but it remains possible that this species is related to one of them rather than other Dendrolimax.

##### Distribution:

Pemba island; probably also East Usambara Mts.

##### Etymology:

*vangoethemi*, a noun in the generative case, for Dr. J. L. van Goethem of IRSNB, in recognition of his thorough and highly accessible monograph on Afrotropical urocyclid slugs.

### Further notes on selected species

#### 3. "Assiminea" aurifera

(Preston, 1912)

Fig. 2

Assimania aurifera Preston 1912: 191–192; pl. XXXI, fig. 9

##### Notes.

Living indiduals were abundant in leaf litter across Pemba, including sites many kilometres from the sea or fresh water (Table 2). As the only African terrestrial assimineid, Verdcourt (2000, 2006) considered Assimania aurifera worthy of a new genus in Omphalotropinae, a group diverse on the Mascarenes and elsewhere (Griffiths and Florens 2006). Assimania aurifera is otherwise recorded only from coastal Kenya and Unguja (Verdcourt 2006). However, its shells are very similar to Assiminia parvula Morelet, 1877, described from Anjouan, Comoros (Morelet 1877). Also terrestrial, Assimania parvula is widespread in the Indo-Pacific and was recorded from Aldabra by Gerlach and Griffiths (2002). Solem (1959) discussed how terrestrial assimineids could be dispersed naturally by sea although we note that Assimania aurifera is a common fossil in Pleistocene deposits in southern Tanzania (Reuter et al. 2010). Further data are needed to resolve this. Note: Both Assimania aurifera and Assimania parvula were described under misspellings of Assiminea Fleming, 1928: Assiminia (Morelet 1877) and Assimania (Preston 1912). The genus Eussoia, to which Assimania aurifera has been referred, now includes only aquatic taxa (Brown 1980).

**Figures 2–12.**
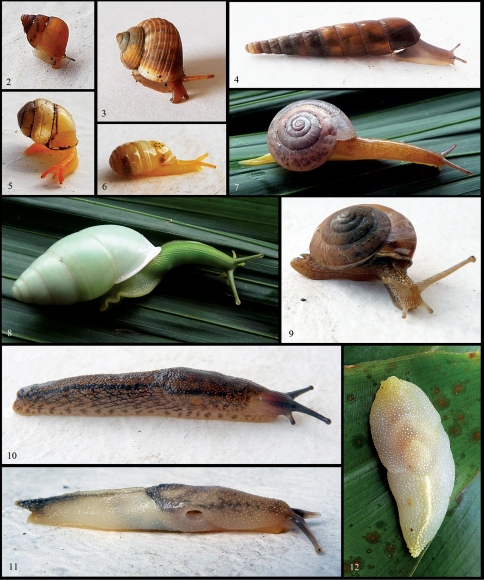
Living animals of selected species from Pemba (not to scale). **2** “Assiminea” aurifera **3** Tropidophora zanguebarica **4** Subulona ordinaria **5** Gonaxis denticulatus **6** Gulella planidens **7** Tayloria shimbiensis **8** Edentulina obesa **9** Trochonanina mozambicensis **10** Pembatoxon insulare **11** “Dendrolimax” vangoethemi (Paratype 2) **12** Dendrolimax vangoethemi (Paratype 1).

#### 4. Cyathopoma azaniense

Verdcourt, 1978

Figs 13–15 

##### Notes.

Cyathopoma azaniense Verdcourt, 1978: 15–16; fig. 1

This species is otherwise known only from the vicinity of Shimoni, Kenya (Verdcourt 1978, 1982), the part of the mainland nearest to Pemba (c. 40km).

**Figures 13–24.**
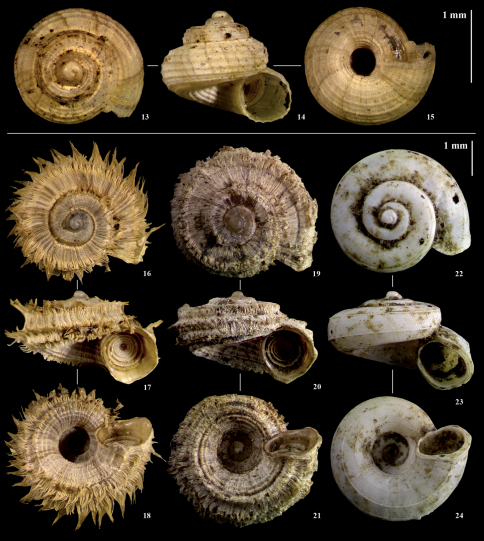
Cyclophoridae from Pemba. **13–15** Cyathopoma azaniense **16–18** Cyathopoma pembense sp. n., live-collected holotype **19–21** Cyathopoma pembense sp. n., paratype (live-collected specimen with periostracal fringe) **22–24** Cyathopoma pembense, sp. n., paratype, dead-collected specimen without periostracum.

#### 5. Tropidophora zanguebarica

(Petit, 1850)

Fig. 3

Cyclostoma zanguebarica Petit de la Saussaye 1850: 53; pl. III, fig. 5

##### Notes.

This group needs revision. Pemba shells are almost identical to those from Jozani Forest, Unguja, including in microsculpture and the range of colour patterns, differing mainly in a smaller maximum size and (Unguja 14.0 × 12.5 mm; Pemba 12.0 × 11.5 mm). There is slight variation in the strength of the spiral raised ridges, though none of the shells are as smooth as *zanguebarica* Petit, 1850 or *letourneuxi* Bourguignat, 1889 in historical collections (e.g. NMW, MNHN). It is hard to know whether this is infraspecific variation or not. Voeltzkow (1923) recorded “Lygatella letourneuxi (Bgl.)” (sic) from Pemba; Haas (1929) recorded it from Chake Chake. As well as *zanguebarica* and *letourneuxi*, Verdcourt (2006) lists two unnamed “species” from “Zanzibar”. Either could correspond to the Pemba taxon; possibly one biological species encompasses all four. One is said to have an affinity to *letourneuxi* and to Tropidophora scabra (H. Adams, 1867), an extinct Mauritian species that varies in sculpture according to Griffiths and Florens (2006). Intriguingly, these authors describe (p.53) a population of a smooth species of Tropidophora being replaced by a rough one over recent decades. Note: Verdcourt treats all East African Tropidophora in subgenus Otopoma Gray, 1850, but the Asian type species of this belongs in Cyclophoridae not Pomatiidae (=Pomatiasidae) (see Neubert 2003).

#### 6. Laevicaulis alte

(Férussac, 1821)

Vaginulus alte Férussac 1821–1822: 14

##### Notes.

Voeltzkow (1923, p. 179) recorded this species from Pemba as “Vaginula brevis” Vaginula brevis Fischer, 1872 is considered a synonym of the widespread Laevicaulis alte (Forcart 1953, Verdcourt 2006). We tentatively refer two small juveniles from Ngezi FR to this species.

#### 7. Gittenedouardia conulina

(von Martens, 1869)

Fig. 29

Buliminus (Pachnodus) von Martens 1869: 153

##### Notes.

The name Gittenedouardia Bank & Menkhorst, 2008 recently replaced Edouardia auctt. non Gude (Bank and Menkhorst 2008). The slender-shelled East African species of Gittenedouardia differ subtly in shell proportions. Two of the Pemba shells are large enough to be adult, at 9.8 × 5.5mm (Fig. 29) and 8.2 × 4.1mm. These were compared with photographs of the lectotypes of Gittenedouardia conulina, Gittenedouardia metula (von Martens, 1895), and Gittenedouardia sordidula (von Martens, 1897) (all in ZMB) and Gittenedouardia tumida (Taylor, 1877) (in BMNH). Gittenedouardia conulina and Gittenedouardia metula appear to be the ends of a shape continuum from slender with less tumid whorls (*conulina*) to broad with tumid whorls (*metula*). The Pemba shells, Gittenedouardia sordidula, Gittenedouardia tumida, the other shells in the Gittenedouardia tumida type lot (see Rowson 2007, p.434), and probably Gittenedouardia metuloides (E. A. Smith, 1899) of Malawi and southern Africa, are each somewhere in the middle. A similar species is recorded from Aldabra (as Edouardia cf. tumida in Gerlach 2006b) and the Comoros Bulimus badiolus Morelet, 1881 also appears to belong to this group. This merits more detailed analysis but for now we associate the Pemba species with Gittenedouardia conulina. This happens to be both the only species previously recorded from Pemba and the oldest available name. It was noted on Pemba by Voeltzkow (1923) and Haas (1929) as Conulinus conulinus (von Martens)

Rowson (2007, p.433–434, 454–455) considered Gittenedouardia conulina one of the few taxa recorded from both Unguja and Pemba, but not the mainland. Given the taxonomic problem this is of little biogeographic significance. Regrettably the confusion in this group may have been added to by contradictorily illustrating Gittenedouardia conulina with a specimen from the mainland (Kilifi, Kenya) (Rowson 2007: Fig. 27). This bleached specimen’s identification (by the late T. Pain) as “Cerastus conulinus (Mts.)” was taken at face value, but it is too large (16.1mm) to be either Gittenedouardia conulina or Gittenedouardia sordidula. The name *sordidula* was introduced by von Martens (1897) to replace the homonym *conulinus* von Martens, 1878 (not von Martens, 1869) after having used the name *conulinus* for what he later considered two separate species in the same genus. As stated above, we consider the lectotypes of these two species, *conulina* and *sordidula* to be very similar, but the drawings in von Martens (1897) rather emphasise the differences which may have helped to mislead Pain. However, whichever species the Kilifi specimen represents, it is not one recorded from Unguja or Pemba.

#### 8. Rhachistia braunsi

(von Martens, 1869)

Fig. 28

Buliminus (Rhachis) von Martens 1869: 150

##### Notes.

All Pemba material appears to be conspecific. Across sites, shells reach only c14mm when adult, and are very thin, not glossy, with faint and irregular spiral and radial striae. One or more brown spiral bands per whorl are present on a weak yellow background, some individuals having a few additional weak brown spots. In several individuals the apex is dark. Multi-banded forms match what (Verdcourt (1961, 2006) calls Rhachidina braunsi var. *quadricingulata* (E. A. Smith, 1890), described from lowland Tanzania. All the Pemba material is here referred to *braunsi*, whose varieties we consider only colour forms. Voeltzkow’s (1923) record of “Rachis brauensis Mart.” (sic) probably refers to Rhachistia braunsi from Fundu I. (Haas, 1929). The genus is here given as Rhachistia rather than Rhachidina (see Solem 1959, Mordan 1992, Herbert and Kilburn 2004).

**Figures 25–32.**
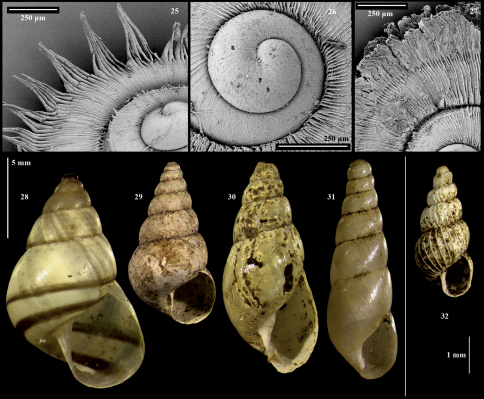
Cyclophoridae and Stylommatophora from Pemba. **25** Cyathopoma pembense sp. n., paratype, periostracal hairs and protoconch of **26** Cyathopoma pembense sp. n., protoconch of paratype with periostracal fringe **27** Cyathopoma pembense sp. n., paratype, periostracal fringe **28** Rhachistia braunsi **29** Gittenedouardia conulina **30** Curvella subvirescens **31** Striosubulina striatella **32** Pseudopeas igembiense.

There is a hypothesis that Bulimus histrio L. Pfeiffer, 1854, described from the New Hebrides, is a synonym of Rhachistia braunsi. Solem (1964) cited Verdcourt (1961) as confirming a suggestion in Solem (1959) that *braunsi* and *histrio* were synonyms, thus proving that *histrio* was an early anthropogenic introduction from East Africa. Though not quite correct (the name *braunsi* did not appear in Solem 1959) this was followed by other workers in the region (e.g. Starmühlner 1970) and there is now a consensus that the Australasian populations originated in East Africa (e.g. Stanisic 1998, Herbert and Kilburn 2004). However, (Verdcourt (1961, 1983, 2006) remained ambiguous about placing the two in synonymy and objected that the colour pattern in Solem’s (1959) black and white picture of a type of Rhachistia histrio had not yet been noticed among East African species. He also noted the existence of other, earlier names. Our material is available should anyone be in a position to resolve this debate.

#### 9. Microcystina minima

(H. Adams, 1867)

Figs 51–53

Macrochlamys minima Adams 1867: 303; pl. 19, fig. 2
Afroguppya rumrutiensis  (Preston, 1911) sensu Rowson 2007 (p. 445, fig. 14)
Dupontia  sp. in Gerlach and Griffiths 2002

##### Notes.

The Pemba material matches that from Unguja figured by Rowson (2007) under the name Afroguppya rumrutiensis, and the “Dupontia sp.” found on Aldabra (Gerlach and Griffiths 2002). Although these three populations appear conspecific, the species is not the mainly East African Afroguppya rumrutiensis. The West African Afroguppya solemi de Winter & van Bruggen, 1992 resembles it closely in size and shape, but has obscure spiral sculpture (de Winter and van Bruggen 1992) whereas these are finely granular. Igor Muratov (pers. comm.; Muratov in press) refers similar material from north-east Mozambique to Microcystina minima, a common species on the Mascarenes (see Griffiths and Florens 2006).

#### 10. Sitala jenynsi

(L. Pfeiffer, 1845)

Figs 36–38

Helix jenynsi Pfeiffer 1845: 131

##### Notes.

Although Voeltzkow (1923) did not mention this species Haas (1929) recorded it from Fundu I. as Trochonanina (Martensia) jenynsi (Pfeiffer) based on Voeltzkow’s material.

**Figures 33–56.**
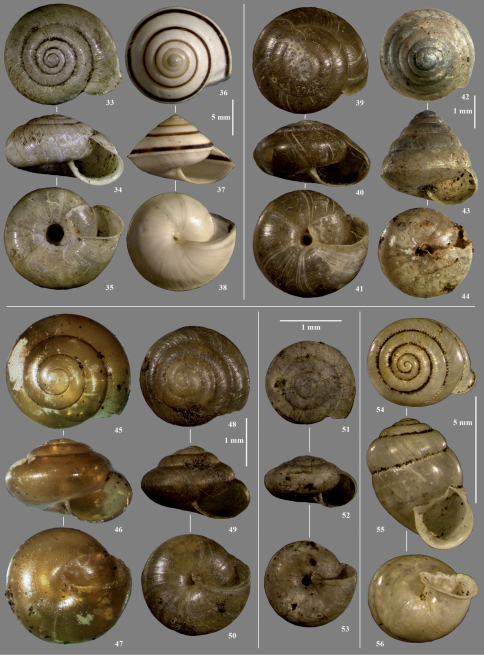
Stylommatophora from Pemba (except where noted). **33–35** Tayloria shimbiense **36–38** Sitala jenynsi (Marafa, near Malindi, Kenya) **39–41** Thapsia insulsa **42–44** Kaliella barrakporensis (Mt. Elgon NP, Kenya) **45–47** Afroguppya quadrisculpta (Udzungwa Mountains NP, Tanzania) **48–50** Afropunctum seminium **51–53** Microcystina minima **54–56** Gonaxis denticulatus.

#### 11. Pembatoxon insulare

van Goethem, 1975

Fig. 10

Pembatoxon insulare van Goethem 1975: 207–216.

##### Notes.

This species was found only in FRs (Table 2) in leaf litter and rotting wood. The only spermatophore obtained was partially digested but the slug recalls Pembatoxon insulare in all other respects including the radula. Van Goethem (1975) noted the holotype (BMNH) was collected in 1901 from “Pemba Island, E. Africa” by C. Crossland but gave no further locality.

Two additional urocyclid slugs were recorded from Pemba by Voeltzkow (1923, p.173) who noted their appearance after heavy rain. These were the only previously recorded speices not relocated in our survey. The difficulties in inventorying slug faunas are well-known (Cameron and Pokryszko 2005) so this does not necessarily imply their absence. They are Elisolimax roebucki (Simroth, 1910) (as Urocyclus roebucki) and Trichotoxon heynemanni Simroth, 1888 (as Trichotoxon voeltzkowi Simroth, 1910).

#### 12. Thapsia curvatula

von Martens, 1897

Thapsia curvatula von Martens 1897: 41; pl. III, fig. 12

##### Notes.

Found only in high forest at Ngezi (Table 2), supporting Verdcourt’s (2000) suggestion that it is a forest specialist.

#### 13. Thapsia insulsa

Preston, 1910

Figs 39–41

Thapsia insulsa Preston 1910: 531; pl. VIII, fig. 14

##### Notes.

The small size, tight coiling, and large umbilicus of this species are distinctive even within this difficult genus (Preston 1910). Verdcourt (2006) records Trichotoxon insulsa from the Shimba Hills (the type locality) and Mrima Hill, and NMW has specimens from Gazi (all localities in coastal Kenya). We found only in non-forest sites (Table 2).

#### 14. Trochonanina mozambicensis

(L. Pfeiffer, 1855)

Fig. 9

Helix mozambicensis Pfeiffer 1855: 91–92; pl. XXXI, fig. 9.

##### Notes.

Occurs in all habitat types (Table 2). Voeltzkow (1923) recorded it from Pemba as “Trochonanina mossambicensis Pts.” and “Trahonemia mossabicensis” (sic), noting numerous individuals. Haas (1929) recorded it as Trochonanina mossambicensis (Pfeiffer) from Chake Chake and Fundu I.

#### 15. Achatina (Lissachatina) allisa

Reeve, 1849

Achatina allisa Reeve 1848–1850: pl. V, fig. 16

##### Notes.

Widespread on Pemba (Table 2). Some individuals from high forest at Ngezi are small (to 61mm high) with a very irregularly thickened outer lip, suggesting adulthood.

#### 16. Achatina (Lissachatina) fulica ?subsp. hamillei

Petit, 1859

Achatina fulica Petit de la Saussaye 1859: 384–5; pl. XIII, fig. 3

##### Notes.

Large shells agreeing with *hamillei* (see Rowson 2007) were the only Achatina found at Msitu Mkuu but apparently did not occur in other FRs (Table 2). Voeltzkow (1923) noted that “Achatina fulica (Fer.)” (sic) was widespread on Pemba; Haas (1929) gave a record from Fundu I.

#### 17. Allopeas gracile

(Hutton, 1834)

Bulimus gracile Hutton 1834: 84, 93

##### Notes.

Pemba specimens reach a large size (13.5 × 3.4 mm, occasionally 15.0 × 3.5 mm, up to 8 whorls), always being relatively narrower than Opeas lamoense. The maximum size is substantially greater than given by most authors (e.g. Gerlach 2006b: 9.4 mm; Griffiths and Florens 2006: 13.5 mm). Said to be restricted to “waste places” by Verdcourt (2000) this species nonetheless occurs in some forest habitats (Table 2). Allopeas gracile is widespread in the tropics but its native range is uncertain. Voeltzkow (1923) noted that “Opea gracile johanninum (Mor.)” (sic) was numerous in rotting logs on Pemba; Haas (1929) gives the record from Chake Chake. Fischer-Piette and Vukadinovic (1974) consider Bulimus johanninus Morelet, 1877, described from the Comoros, a synonym of Allopeas gracile. Recent authors (e.g. Neubert 1998, Gerlach 2006b, Griffiths and Florens 2006) rather than Verdcourt (2006) and Rowson (2007) are followed here in treating this species in Allopeas as distinct from Lamellaxis.

#### 18. Curvella subvirescens

(E. A. Smith, 1890)

Fig. 30

Bulimus (Hapalus) subvirescens Smith 1890: 156; pl. 5, fig. 12

##### Notes.

This species reaches 12.0 × 5.0 mm and is relatively broader than Pemba’s other subulinids. It was found only in high moist forest at Ngezi (Table 2). It keys out to Curvella subvirescens using Verdcourt’s (2002) key and resembles the types at BMNH. Verdcourt (2006) records Curvella subvirescens from the Nguru Mts. and Uluguru Mts. and notes its similarity to the Tanzanian Curvella sinulabris (von Martens, 1878) and Kenyan Curvella pertranslucens Preston, 1910, the latter described from the Shimba Hills (Preston 1910).

#### 19. Opeas delicatum

Taylor, 1877

Opeas delicatum Taylor 1877b: 281–282; pl. III, fig. 3

##### Notes.

Pemba specimens reach 7.0 × 2.5 mm, being much smaller and narrower than Opeas lamoense. Griffiths and Florens (2006) figure specimens referred to Allopeas clavulinum (Poitiez & Michaud, 1838) and Allopeas mauritianum (L. Pfeiffer, 1847) that they suggest were introduced to the Mascarenes from East Africa. Both resemble some specimens of Opeas delicatum. Verdcourt was familiar with Allopeas clavulinum in botanic gardens in the UK, which in turn have been said to come from East Africa (Kerney and Cameron 1979) yet never included Allopeas clavulinum in his East African lists (Verdcourt 1983, 2000, 2006). This should be further investigated.

#### 20. Opeas lamoense

Melvill & Ponsonby, 1892

Buliminus lamoense Melvill and Ponsonby 1892: 90; pl. V, fig. 12

##### Notes.

Pemba specimens reach 11.0 × 4.0 mm, being relatively much broader and with a relatively larger body whorl than Allopeas gracile. At one high forest site (Ngezi N2) some individuals have much stronger ribs, although the shell shape is similar.

#### 21. Striosubulina striatella

(Rang, 1831)

Fig. 31

Helix (Cochlicope) Rang 1831: 34–35; pl. III, fig. 7

##### Notes.

Found only at Mtondoni (Table 2). The genital anatomy conforms exactly with that of Striosubulina striatella as figured by Schileyko (1999), though the bursa copulatrix is less voluminous. A tropical West African species, Striosubulina striatella has been widely introduced including to the Mascarenes (Griffiths and Florens 2006). This appears to be the first record from East Africa.

#### 22. Subulina octona

(Bruguière, 1789)

Bulimus octona Bruguière 1789: 325

##### Notes.

The geographical origin of this species is unknown. Gerlach (2006b) notes it has been found as a subfossil on Aldabra but Griffiths and Florens (2006) suggest it is originally Neotropical. Verdcourt (in litt. 2006) noted that he knew of no material of Subulina octona from East Africa so it is possible the species is spreading. The date of authorship follows Bank and Menkhorst (2008).

#### 23. Subulona ordinaria

(Preston, 1910)

Fig. 4

Homorus ordinaria Preston 1910: 534; pl. IX, fig. 25

##### Notes.

Verdcourt (2000) treated this as a forest species but it also occurs in other habitats on Pemba (Table 2). It is known from the Shimba Hills (Preston 1910; type locality) and from the Sigi Valley in the East Usambara Mts. (Verdcourt 2006).

#### 24. Edentulina obesa

(Taylor, 1877)

Fig. 8

Buliminus obesa Taylor 1877a: 255; pl. II, fig. 3

##### Notes.

This species was previously recorded from Pemba by (Verdcourt (1983, 2006).

#### 25. Gonaxis (Gonaxis) denticulatus

(Dohrn, 1878)

Figs 5 54–56

Streptaxis denticulatus Dohrn 1878: 152

##### Notes.

This species is widespread and abundant on Pemba (Table 2). The specimens are assigned to the widespread Gonaxis denticulatus pro tem. rather than the more restricted Gonaxis gibbonsi Taylor, 1877. A revision of the East African taxa attributed to “Gonaxis” is currently under way (Rowson in prep.).

#### 26. Tayloria shimbiensis

Connolly, 1922

Figs 7 33–35

Tayloria shimbiensis Connolly 1922: 487

##### Notes.

This species has not previously been recorded beyond the type locality (Shimba Hills) (Verdcourt 2006). According to Verdcourt (1958), its shell differs from that of the other known lowland species, Tayloria helicoides (C. R. Böttger, 1913) only in relative spire height and strength of sculpture. The latter is known only from Kipatimu, Kilwa District, south of the Rufiji Delta.

#### 27. “Gulella” (Aenigmigulella) aenigmatica

(E. A. Smith, 1890)

Figs 57–59

Ennea aenigmatica Smith 1890: 164; pl. 6, fig. 11

##### Notes.

Pemba material matches Usambara material (NMW) of this Eastern Arc species very well. No species of Aenigmigulella has previously been reported from the coastal region (Verdcourt 2000, 2006) and it is unknown from Unguja. If native to Pemba it would thus suggest a special Eastern Arc affinity. However we note it was not found in forest reserves apart from a juvenile at Msitu Mkuu FR (Table 2). On Pemba, juvenile shells have complex dentition at a variety of stages. The living animal is cream-coloured. Recent systematic work (Rowson 2010) indicates “Gulella” aenigmatica does not belong in the genus Gulella L. Pfeiffer and a genus-level revision is in progress.

**Figures 57–63.**
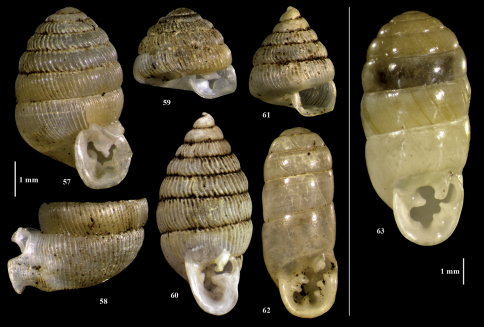
Streptaxidae from Pemba. **57–58** “Gulella” (Aenigmigulella) aenigmatica **59** “Gulella” (Aenigmigulella) aenigmatica, juvenile **60** “Gulella” radius **61** “Gulella” radius, juvenile **62** Gulella gwendolinae (resembling var. *scissidens*) **63** Gulella planidens.

#### 28. "Gulella" radius

(Preston, 1910)

Figs 60–61

Ennea radius Preston 1910: 529; pl. VII, fig. 8

##### Notes.

As noted by Verdcourt (1985) there is either substantial shell variation in the species Ennea radius Preston, 1910, or it comprises a complex of related taxa. This cannot be resolved without a thorough revision. Preston’s (1910) type from the Shimba Hills is said to be 3.25mm high and is strongly acuminate. Verdcourt (1985) figured a specimen from Diani Beach, Kenya which is much less acuminate but measures 4.23mm (calculated from his drawing). At up to 5.45mm, Pemba specimens are larger still, but resemble the type in being strongly acuminate and with less tumid whorls than the Diani Beach material. Specimens referred to Gulella radius from Unguja (Rowson 2007) are small (to 3.0mm) and not strongly acuminate. The peristomal teeth are in the same basic pattern in each of these populations, but vary in their size and complexity, none being quite as different as some of the other nominal species (discussed in Rowson and Lange 2007). What does appear relatively constant is the size, shape, and sculpture of Pemba specimens, which occur throughout the island (Table 2). They may form an island taxon worthy of subspecies or species status, which is given consideration in the discussions on endemism in the present paper. Recent systematic work (Rowson 2010) indicates “Gulella” radius does not belong in the genus Gulella L. Pfeiffer and a genus-level revision is in progress.

#### 29. Gulella gwendolinae

(Preston, 1910)

Fig. 62

Ennea gwendolinae Preston 1910: 527; pl. VII, fig. 3

##### Notes.

Six of the seven adults from Misali Island have an additional palatal tooth, recalling var. *scissidens* Connolly, 1922, described from Dar-es-Salaam. The additional tooth is not present on either of the two adults from Ras Kiuyu. This is a very widespread species and several such forms have been named. Neubert (1998) points out a discrepancy between Paladilhe’s (1872) description and figure of Ennea isseli Paladilhe, 1872 from Yemen, and figures an additional specimen which strongly resembles Gulella gwendolinae. This raises the possibility that Ennea isseli is a senior synonym of Gulella gwendolinae and, if so, also the question of whether it is truly native to Arabia. A direct comparison of types is advised.

#### 30. Gulella planidens

(von Martens, 1892)

Fig. 63

Ennea planidens von Martens 1892: 179

##### Notes.

This species, widespread in East and South-east Africa (van Bruggen and van Goethem 1997) was much more frequent in non-forest habitat types than in forest (Table 2). Voeltzkow (1923) recorded “Gulilla laevigata (Dohn)” (sic); Haas (1929) recorded Gulella laevigata (Dohrn) from Chake Chake. The record probably actually refers to Gulella planidens (see van Bruggen and van Goethem 1997).

**Figures 64–75.**
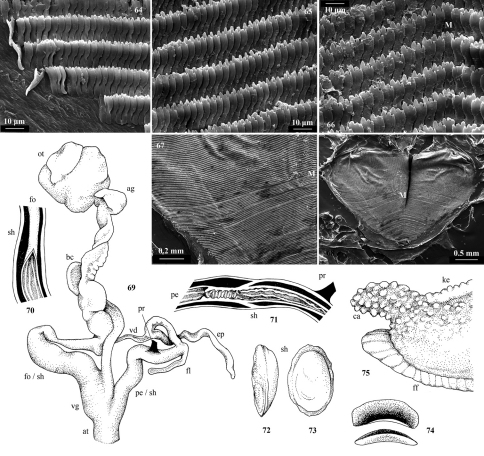
“Dendrolimax” vangoethemi sp. n. from Pemba. **64–68:** Radula of holotype: **64** marginal teeth **65** lateral teeth **66** central teeth including median tooth (M) **67** whole radular row **68** whole radula. **69–71** genitalia of Paratype 2: **69** genitalia **70** free oviduct and sheath **71** penis and sheath; **72–73** shell of holotype: **74** jaw of holotype **75** tail of Paratype 2 in 80% ethanol. Abbreviations: **ag** albumen gland **at** atrium **bc** bursa copulatrix **ca** caudal appendage **ep** epiphallus **ff** foot fringe **fl** flagellum **fo** free oviduct **ke** keel **M** median radular tooth **ot** ovotestis **pe** penis **pr** penial retractor **sh** sheath **vd** vas deferens **vg** vagina.

## Discussion

### a) Impoverishment and community diversity

Though the number of species varies between FRs and habitat types, the total number of species for Pemba is not low. Richness values for each of the FRs are within the range for those of moderately rich to rich coastal forests in the region (18–50 species) (Emberton et al. 1997, Tattersfield 1998, Lange and Mwinzi 2003, Rowson 2007). Emberton et al. (1997) found northern Tanzanian coastal forests were richer than southern ones. They found Amboni caves (due west of Pemba on the mainland, at less than 100m elevation) to be the richest site of all with 50 species; Tattersfield (1998) also found it to be the richest but recorded only 29 species. The difference probably partly reflects taxonomic discrepancies in the morphospecies approach. At least 29 species are recorded from Jozani Forest on Unguja (Rowson 2007). When both habitat types at Ngezi FR are combined, the total of 33 species makes it one of the richest of all East African coastal forests, exceeding some with a much larger area including Arabuko-Sokoke Forest in Kenya (25 species; Lange and Mwinzi 2003). When the two habitat types at Ngezi FR are considered individually, they are still relatively rich (27 on sand/soil and 25 on coral rag). Thus each of Pemba’s FRs supports a fauna of typical richness for northern coastal Tanzania, while Ngezi is especially rich, meaning none are strongly impoverished relative to forests on the mainland. The total recorded fauna of Pemba itself (49 species) is approximately 15% lower than that for Unguja (58 species) so appears slightly impoverished overall. However, the latter is a slightly larger island and has received far more collecting attention historically. There is no data from Mafia or from a comparably-sized area of lowland habitat from the mainland apart from Arabuko-Sokoke. There, in a total area of 372 km2 consisting of several forest habitat types, 1263 individuals (comparable to the total in our survey) were recorded representing just 25 species (Lange and Mwinzi 2003). Thus we conclude that there is no good evidence that Pemba’s land-snail fauna is impoverished.

The contrast in species composition between habitat types at Ngezi FR shows the importance of its diversity of habitats (cf. Beentje 1990). The apparent difference in abundance could be explained by the apparently rapidly draining sandy soil reducing available moisture. Alternatively, soil pH (the only factor found to significantly influence land-snail abundance at Arabuko-Sokoke; Lange 2003) could explain the difference. Species richness is low to intermediate outside FRs, indicating non-forest habitats are suitable for at least part of Pemba’s land-snail fauna. This includes several species previously characterised as forest specialists by Verdcourt (2000). Unless this simply reflects a lack of information available to Verdcourt (2000), it suggests that Pemba’s woody cultivation and moist climate might permit locally-adapted species to persist in a broader range of habitats than their mainland counterparts. A similar observation has been made for Pemba’s endemic birds (Catry et al. 2000).

### b) Imbalance

Across land-snail families, species were recorded in the same rank order and approximately the same proportions as on Unguja (Rowson 2007) and the coastal region as a whole (Verdcourt 2000). Emberton et al. (1997) gave figures on these proportions for coastal forests; though not strictly comparable because of taxonomic discrepancies, the rank order of the three main families is the same and the proportions similar for most forests. The major part of the fauna thus provides no strong evidence of imbalance compared with neighbouring continental areas.

The absence of Maizaniidae (i.e. Maizania) is worthy of comment. We are unlikely to have overlooked the durable, conspicuous and often abundant shells of this group at our sites. It occurs in suitable habitat throughout East Africa including Arabuko-Sokoke and the Usambaras; one species is known from the coast, and one from Unguja (Lange and Mwinzi 2003, Verdcourt 2006, Rowson 2007). However, Maizania is absent from the Pliocene-Pleistocene central highlands of Kenya, a montane forest area of apparently suitable habitat (Verdcourt 1984). Its absence from Pemba could suggest Pemba, unlike Unguja, was isolated before Maizania could reach it. Alternatively, Maizania may have reached Unguja only after it became an island, in which case its failure to reach Pemba would have to be explained by stronger isolation by the Pemba Channel. Beyond suggesting the two islands were isolated independently, which clashes with the distribution of Pembatoxon insulare (see below), the absence of Maizania does not resolve the question of oceanicity.

### c) Endemism and affinities

The rate of endemism (4–8% of species) is very similar to that on Unguja. There, three species (5% of a total of 58) were considered endemic by Rowson (2007) who considered this a rate comparable to coastal forests on the mainland. There are doubts over the species status of several of these taxa, so interpretations of 8% for Pemba vs. 0% for Unguja, or 2% for Pemba vs. 5% for Unguja are possible but, in our opinion, not justified if the margins of uncertainty in taxonomy are taken into account. There are no endemic genera or subgenera. Species with clear affinities to more distant faunas, e.g. Madagascar, the Mascarenes or Asia have been noted in other groups on Pemba (e.g. Moreau and Pakenham 1940, Beentje 1990, Dijsktra et al. 2007). The endemic species have apparently close relatives on the Comoros, but also on the mainland, so are not unusual among Pemban taxa or indeed the rest of the Tanzanian coastal fauna (Rowson 2007, and unpubl. obs.). The relict Gonospira expatriata Preston, 1910, which has apparent Mascarene affinities, and was (or is) extant in coastal Kenya (Verdcourt 2000) has not been found on Pemba. Unless the island once supported additional endemics that are now extinct, these patterns argue against a long history of isolation, especially one many times longer than that of Unguja. We admit to finding this surprising in the light of other taxa reported from Pemba and the debate over its isolation. It is possible that Pemba’s land-snail fauna has suffered disproportionately from post-isolation extinction e.g. by drought, fire, tsunami, or sea-water inundation, explaining the lack of endemics. Recent work indicating a rapid uplift by 80–110m in parts of coastal Tanzania over the last 44,000 years (Reuter et al. 2010) raises the question of whether part or all of Pemba subsided below sea level before this time and was subsequently reuplifted. However, sea level itself was 50m or more lower than present during this period (see Reuter et al., 2010) and inundation would not explain the survival of endemics in other taxonomic groups.

Conversely, 92–98% of Pemba’s land-snail species occur elsewhere. To date about 8 of these (16%) are known only from small areas of adjacent Tanzania or Kenya (Cyathopoma azaniense, “Gulella” aenigmatica, Tayloria shimbiensis etc.) and could comprise a vicariant fauna whose ranges were split only by the Pemba Channel graben. Alternatively, these and the remainder that occur more widely (Gulella planidens, Streptostele acicula, etc.) could have arrived by post-isolation dispersal, with species occurring nearby most likely to arrive soonest. Successful dispersal to Pemba argues against an especially strong isolation, since gene exchange with the mainland would remain possible. This contrasts with Pemba’s volant species for which winds are thought to have strongly limited westward passage from the mainland (Moreau and Pakenham 1940; Baker and Baker 2002). Although accidental introduction by man has played a largely unknown but probably greater part in the land-snail fauna, subfossils on Aldabra (e.g. Gerlach and Griffiths 2002) indicate natural, overseas dispersal by land-snails in the region. Rivers outflowing eastwards from the mainland (e.g. the Pangani, Wami and Ruvu) could aid the dispersal of rafting taxa such as land-snails to the islands, even against seasonally prevailing currents. This could explain the discrepancy with the endemism in volant taxa.

At least 70% of Pemba’s fauna is shared with Unguja. Two taxa (4% of Pemba’s fauna, or 3.5% of Unguja’s) appear to be restricted to both islands so could signal a recent connection or successful post-isolation dispersal between them. However, the slug Pembatoxon insulare is the only well-characterised species of the two, and is identifiable with certainty only from spermatophores, so may yet have been overlooked on the mainland. This seems inconclusive evidence on which to propose a vicariant relationship between Unguja and Pemba, while at least one absence (Maizania) suggests they were isolated independently.

## Conclusion

Pemba’s fauna as revealed by our survey shows no unequivocal evidence of impoverishment, imbalance, or a high rate of endemism so appears little or no more oceanic than Unguja’s. Two land-snail species distributions that might result from older vicariance give conflicting signals: that of Maizania suggests the islands were isolated independently, while that of Pembatoxon insulare suggests they were not. Therefore, although various interpretations are possible, the current consensus from geological data that Pemba has been isolated for much longer than Unguja is not reflected in the snail fauna of the two islands. This phenomenon seems most likely explained by the Pemba channel being a weak barrier to land-snail dispersal, which might explain the discrepancy with the endemism rate of volant taxa. Nonetheless, in the light of our results a critical re-examination of the geological data on the formation of the Pemba channel – especially the timing of the graben faulting and the rate of land subsidence – would be worthwhile. Despite Pemba’s snail fauna lacking the signature of a long period of isolation, this island, and in particular its three FRs, does support endemic land-snail species, and several otherwise found only in small areas of the mainland. This makes its fauna of global conservation importance. Evidence of at least one introduction not yet noted elsewhere in East Africa gives some cause for concern. Moreover, the discovery of undescribed taxa suggests much remains to be learnt about land-snails in this region of endemism.

## Supplementary Material

XML Treatment for Cyathopoma pembense

XML Treatment for "Dendrolimax" vangoethemi

XML Treatment for "Assiminea" aurifera

XML Treatment for Cyathopoma azaniense

XML Treatment for Tropidophora zanguebarica

XML Treatment for Laevicaulis alte

XML Treatment for Gittenedouardia conulina

XML Treatment for Rhachistia braunsi

XML Treatment for Microcystina minima

XML Treatment for Sitala jenynsi

XML Treatment for Pembatoxon insulare

XML Treatment for Thapsia curvatula

XML Treatment for Thapsia insulsa

XML Treatment for Trochonanina mozambicensis

XML Treatment for Achatina (Lissachatina) allisa

XML Treatment for Achatina (Lissachatina) fulica ?subsp. hamillei

XML Treatment for Allopeas gracile

XML Treatment for Curvella subvirescens

XML Treatment for Opeas delicatum

XML Treatment for Opeas lamoense

XML Treatment for Striosubulina striatella

XML Treatment for Subulina octona

XML Treatment for Subulona ordinaria

XML Treatment for Edentulina obesa

XML Treatment for Gonaxis (Gonaxis) denticulatus

XML Treatment for Tayloria shimbiensis

XML Treatment for “Gulella” (Aenigmigulella) aenigmatica

XML Treatment for "Gulella" radius

XML Treatment for Gulella gwendolinae

XML Treatment for Gulella planidens
